# Total titratable acidity and organic acids of wines produced from cactus pear (*Opuntia‐ficus‐indica*) fruit and Lantana camara (*L. Camara)* fruit blended fermentation process employed response surface optimization

**DOI:** 10.1002/fsn3.1745

**Published:** 2020-07-02

**Authors:** Zenebe Tadesse Tsegay

**Affiliations:** ^1^ College of Dryland Agriculture and Natural Resources Department of Food Science and Post‐Harvest Technology Mekelle University Mekelle 231 Ethiopia

**Keywords:** cactus pear, Lantana camara fruit, organic acid, response surface optimization, wine fermentation

## Abstract

Fruits and fermentation methods are important sources of organic acids that determine organoleptic properties, microbiological and biochemical stability of fruit wines. This study is aimed at investigating total titrable acidity and organic acids of fruit wines produced by response surface optimization of cactus pear and *Lantana camara* fruits blend and cactus pear fruit alone. The predictive mathematical model of the blended fruit wine is well fitted (*R* ^2^ = 0.9618 and absolute average deviation (AAD) = 2.06%). The optimum values of fermentation temperature, inoculum concentration, and *Lantana camara* fruitjuice concentration to produce predictive total titrable acidity of 0.8% (w/v citric acid) were 24°C, 10% (v/v), and 10.7% (v/v), respectively. The blended fruit wine was with lower total titrable acidity (w/v citric acid) of 0.83 ± 0.058% compared to wine produced from cactus pear fruit alone 1.06 ± 0.27%. The high performance liquid chromatography (HPLC) analysis of both produced wines revealed the difference in concentration of citric (±3.35 mg/ml), L‐tartaric (± 3.71 mg/ml), and L‐ascorbic acid (± 0.07 mg/ml). Citric acid was predominant organic acid in both fruit wines, and its content in the cactus pear is 7.09 ± 0.07 mg/ml and blended fruit wine 4.74 ± 0.07 mg/ml.

## Graphical abstract

RSM optimization of cactus pear and Lantana camara fruits blend for wine production. TTA of the produced blended fruit wine predicting model is accurate. HPLC analysis of organic acids of the produced wine prevailed the presence of citric, tartaric, and ascorbic acids.

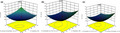

## 1 INTRODUCTION

Fruits contain organic acids which are main source of fermented food production. Fruit wine fermentation process change diversity of the wine color, aroma, pH, microbial stability, and antioxidant capacity with acidity of the final wine (Coelho et al., 2015). Total acidity, titrable acidity, and volatile acidity of a fruit wine are originated from the type of organic acid present. Total acidity and titrable acidity are chemically different due to electronic structure variability of these organic acids. Total acidity is the property used to express proton equivalence of the organic acid anions, and the number of protons of all undissociated organic acids would contain the fruit wine. The measured value indicates both the dissociated and undissociated forms of each individual acid. It excludes carbonic acid (due to carbon dioxide) and sulfur dioxide content. Conversely, titrable acidity indicates the number of dissociated protons, from organic acids, which are neutralized by a strong base during titration, always less than total titratable acidity. Moreover, volatile acids of the acetic acid series present in fruit wines as free form or as combined salts uses to express the volatile acidity (Darias‐Martín, Socas‐Hernández, Díaz‐Romero, & Díaz‐Díaz, 2003; Dias, Duarte, & Schwan, 2017). In a study reported by Zhong, Chen, and Yang &Li (2020), Kiwifruit wine fermentation shown degradation of citric, malic, and tartaric acids which accounts 74.13%, 18.41%, and 3.65% of total acid to 69.49%, 12,76%, and 2.59% of total acid correspondingly after fermentation. Total titratable acidity and organic acids (malic acid, citric acid, and lactic acid) present in fruit wine are mainly responsible to sour taste (Lee et al., 2013). Volatile acids in wines account 90% of the content of acetic acids which can originate from some fruits and also can be synthesized by yeasts during fermentation (chemical oxidation of ethanol in the presence of oxygen). Similarly, acetic acid bacteria can synthesize acetic acid during wine aging and storage (Dias et al., 2017). Hence, fermentation process should optimize total titratable acidity as well as contribution of individual organic acids to assure the overall quality profile of the wine.

Organic acids next to sugars significantly present in fruit wines are major wine chemistry descriptors. Basic fruit wine qualities such as sensory, antioxidant capacity, antimicrobial activities, pH, and color are dependent on the amount and types of organic acids available (Vilela, 2019). Major fixed organic acids such as L‐tartaric (citrus‐like taste), L‐malic (metallic, green‐apples taste), L‐lactic (sour and spicy), citric (fresh and pleasant citrus‐like taste), and succinic (Sour, salty, and bitter taste) contribute different sensory properties on fruit wines. Similarly, major volatile organic acids such as acetic acid enable fruit wine to show vinegar‐like taste which indicates poor quality of the produced fruit wine (Vilela, 2019). Citric acid as weak organic acid can bind with calcium oxalate crystal surface which inhibits urinary crystallization and stone formation. This property indicates that intaking appropriate (excessive intake may cause hypocalcemia) citric acid enable to protect against the development of diabetic complications (Zhong et al., 2020). It shows also significant antioxidant capacity by chelating metal ions which prevents from browning of the fruit wine. Citric acid is potent in antimicrobial activity against development of unwonted molds and bacteria during fruit fermentation and wine stabilization (Vilela, 2019). Unlike tartaric, citric acid is potential intermediate of tricarboxylic acid cycle (TCA cycle) which occurs in the metabolism of almost every organism. Ascorbic acid composing fruit wine has improved antioxidant property. Wine produced from cactus pear fruit antioxidant concentration measured as 235.3 mg/L equivalent to ascorbic acid (Zenebe, Chanukya, & Solomon, 2018). Organic acids lower pH of fruit wines which prevents microbiological development during wine aging. Furthermore, wine bouquet is formed due to the chemical reactions of organic acids during aging (Tasev, Stefova, & Ivanova, 2016). Therefore, optimizing concentration of total titratable acidity and organic acids originate from the fruit nature as well as produced during the alcoholic fermentation process is mandatory to ensure organoleptic properties, microbiological, and biochemical stability of fruit wines.

Cactus pear fruits are considered as one of the nutritious fruits. Orange types of cactus pear fruits (spiny *Opuntia ficus‐indica* verities) grown in Adigrat, Tigria, Northern Ethiopia, are known with fruit description as ovoid shape, medium (120–150 g) size, flatten scar position of receptacle, yellow‐orange peel color, yellow‐orange pulp color, and firm pulp (Tesfay, Mulugeta, & Tadesse, 2011). Seeds of prickly pear (*Opuntia ficus‐indica*) are rich in mineral contents (Ca, Cu, Fe, K, Mg, Na, P, Mn and Zn) as well as palmitic, oleic, and linoleic acids (Al‐Juhaimi & Özcan, 2013; Matthäus & Özcan, 2011; Özcan & Al Juhaimi, 2011). Lantana camara plants are flowering ornamental shrub with worldwide distribution in subtropical, tropical, and temperate climates. Its ripen fruit has dark purple color. Ingestion of these ripen (including unripe) fruit berries has no associated health effects (Carstairs, Luk, Tomaszewski, & Cantrell, 2010). Lantana camara plants are rich in polyphenols having highest potential as free radical scavengers (Sousa, Rocha, Barros, Barros, & Costa, 2013). Natural fermentation of red cactus pear juice (*Opuntia streptacantha*) has been applicable to produce wine with attractive color, flavor, and taste qualities (Navarrete‐Bolaños et al., 2013). Fermented cactus pear fruit juice has shown health‐promoting properties and enhanced antioxidant activities (Verón et al., 2019). Cactus pear and Lantana camara fruits contain total titrable acidity 0.10 ± 0.01% (w/v of tartaric acid) and 0.13 ± 0.01% (w/v of tartaric acid), respectively. The predominant organic acid determined in each of these fruits is tartaric acid (Zenebe & Kidu, 2019). Analysis of ascorbic acid content from different ripen prickly pear (*O. ficus‐indica* L.) fruits collected from five different locations of Turkey was reported from 124.82 mg/kg to 240.25 mg/kg (Belviranlı et al., 2019). Besides, fermentation process of blended cactus pear and Lantana camara fruits using *Saccharomyces cerevisiae* produced wine with total titrable acidity and total organic acids of 0.33 ± 0.01% (w/v of citric acid) and 5.57 ± 0.14 mg/ml, respectively, in which citric acid is a predominant acid (Zenebe & Kidu, 2019). Navarrete‐Bolaños et al. (2013) reported that young wine produced from cactus pear fruit contained oxalic, tartaric, malic, lactic, and acetic which elevated the wine acidity. Mixing of fruits, addition of medicinal herbs, and blending ginger extract during fermentation process enhanced organoleptic properties, microbiological, and biochemical stability of the produced fruit wines (Lee et al., 2013; Ogodo, Ugbogu, Ugbogu, & Ezeonu, 2015; Yusufu, Pg, & Sa, 2018; Zenebe & Kidu, 2019). According to the survey study reported by Chidi, Bauer, and Rossouw (2018), there is a knowledge gap in the influence of individual wine yeast fermentation to the total organic acid profile of fruit wines. Sensory attributes of the wines are affected by these acids with descriptors ranging from fresh to sour to metallic. Similarly, sensory quality is strongly correlated with the sugar–acid balance of the fruit wine. Limited fruit wine fermentation process was investigated to show the effect of fermentation variables (fermentation temperature, pH, inoculum concentration, ratio of mixing substrates) on total titrable acidity and organic acid content of the intended wine. Despite the application of optimization methods to control alcohol, total phenol, antioxidant, and sensory qualities, controlling total titrable acidity and organic acid quality profiles of the produced fruit wine have limited studies (Coelho et al., 2015; Nikhanj & Kocher, 2018; Peng, Lei, Zhao, & Cui, 2015; Zenebe & Solomon, 2020).

Response surface method coupled with composite central rotatable design (CCRD) is appropriate for optimization of fruit wine fermentation process determining input factors (Nikhanj & Kocher, 2018; Peng et al., 2015; Zenebe et al., 2018; Zenebe & Solomon, 2020). Organic acid composition of fruits as well as fermentation process input factors directly and jointly affects total titrable acidity of the final wine. Thus, controlling and developing fruit fermentation process predictive mathematical model for better organoleptic properties, microbiological, and biochemical stability is vital. In the current study, fermentation process parameters (fermentation temperature, inoculum concentration, and Lantana camara fruit juice concentration) of blended cactus pear and Lantana camara fruits were optimized considering total titrable acidity of the final wine. Total titrable acidity and organic acid composition of wines produced at this optimized fermentation process in comparison with the wine produced from cactus pear fruit alone were investigated.

## 2 MATERIALS AND METHODS

### 2.1 Chemicals

L‐tartaric acid, L‐ascorbic acid, citric acid, and oxalic acid were purchased from Wise team PLC chemical reagent (Addis Ababa, Ethiopia) originated from UNI‐CHEM, India. Yeast (*Saccharomyces cerevisiae*) was purchased from a supermarket, Addis Ababa, Ethiopia. All chemicals and solvents used in this study were of analytical grade and used as supplied.

### 2.2 Fruit samples collection and preservation

Lantana camara (*L. Camara,* voucher specimen number of ET001) fruits with dark purple color were collected using dry polyethylene bag from Axum, Ethiopia, during March and April (Tadesse, Engidawork, Nedi, & Mengistu, 2017). The healthy ripened Lantana camara berries were sorted, destemmed, and washed by immersing in 3 L distilled water containing plastic jar. Orange types of cactus pear fruits previously identified by Tesfay et al. (2011) (Collection number: TOfi‐1 and variety: spiny *Opuntia ficus‐indica*) were collected from farmers during the peak production time of April and July, Adigrat, Ethiopia. The healthy matured cactus pear fruits were preserved using icebox (at about 10°C) during three hours transportation (to Chemistry Laboratory, Aksum University), sorted, and washed with running tap water. The washed and properly drained both fruit types were stored at 4°C until fruit juices were extracted.

### 2.3 Juice preparation of cactus pear and Lantana camara fruits

Domestic juicer machine (Electric Juicer, BL‐727, Japan) was used to extract both fruit type juices. During the Lantana camara fruit juice extraction, 1 L distilled water was added to enhance the fruit juice extraction. Then, 70% w/v of juice was produced. Cactus pear fruits were manually peeled to remove its outer coat, and the edible part of the pulp was chopped up. To prevent prefermentation as well as microbial contaminations, both juice types were preserved by adding 70 mg/L sodium thiosulfate. Finally, extracted juice types were preserved at 4ᵒC until filtered using sterilized cotton cloth mesh to remove the seeds and fibers (Zenebe & Kidu, 2019; Zenebe & Solomon, 2020).

To consider the fruit types quality as well as the requirement of additional fermentation substrate adjustments and nutrient addition, both extracted juices of fruit type were chemically characterized which is reported in our previous study (Zenebe & Kidu, 2019).

### 2.4 Preparation of yeast culture

Biomass of the yeast cell (1.7x10^6^ CFU/mL) was developed first by inoculating into 50 ml of sterilized YEPD media (1% m/v yeast extract, 2% m/v peptone, and 2% m/v glucose) and then transferring into sterilized cactus pear juice pulp adjusted at pH of 3.4 and sugar content of 200 g/L as briefly described by Zenebe and Kidu (2019). The YEPD media were incubated for 24 hr with 120 rpm shaking speed at 28°C, and the cactus pear juice biomass was incubated for 36 hr with a shaker speed of 150 rpm at 28°C. The yeast biomass was used directly for wine fermentation inoculation after incubation.

### 2.5 Fruits fermentation process and stabilization of the produced wines

Cactus pear and blended fruit wines were produced separately to investigate the effect of Lantana camara fruit blending effect on total titratable acidity as well as organic acid properties of booth final wines. As Zenebe and Kidu (2019) described the procedure briefly, the 350 ml extracted juice samples were placed into 500 ml glass flasks fitted with venting plastic valve airlocks, adjusted to the sugar content of 200 g/L (expressed by D‐glucose) using table sugar, to pH 3.9 using 0.5 g/L tartaric acid solution and the fermentation process was carried for 6 days by shaking twice a day for the first three days and once for the remaining fermentation days. Free runs of the fermented products were filtered from each sample run using sterilized cotton cheese close. Each sample was packaged in a sterilized brown 330 ml glass bottles. Finally, each wine samples were pasteurized at a temperature of 65ᵒC for 20 min and preserved by adding 70 mg/L SO_2_ for twenty days at room temperature (24ᵒC).

#### 2.5.1 Wine production from cactus pear only

To study the total titratable acidity and organic acid composition as well as to show the effect of Lantana camara fruit blending, cactus pear fruit wine was produced using the optimized fermentation developed by Zenebe et al. (2018). The fruit fermentation substrate was adjusted at pH of 3.4 and 16% inoculum concentration and fermented at of 30 *◦*C temperature.

#### 2.5.2 Wine production from cactus pear and Lantana camara fruits blend

Blended fruit wine was produced (cactus pear with Lantana camara fruits) using the experimental design developed in Table 1. The input factors fermentation temperature, inoculum concentration, and Lantana camara fruit juice concentration (70% w/v juice extract) were adjusted according to the final experimental design shown in Table 1.

**Table 1:** Central composite rotatable experimental design (CCRD) of input factors with actual and predicted response values

Std	Temperature (°C)	Inoculum Concrntration (% v/v)	Lantana camara fruit concentration (%v/v)	Measured Total titratable acidity (%, w/v)	Predicted Total titratable acidity (%, w/v)
1	20(−1)	8(−1)	8(−1)	1.1	1.11
2	30(+1)	8(−1)	8(−1)	1.15	1.21
3	20(−1)	12(+1)	8(−1)	1.18	1.13
4	30(+1)	12(+1)	8(−1)	1.74	1.67
5	20(−1)	8(−1)	12(+1)	1.15	1.24
6	30(+1)	8(−1)	12(+1)	0.95	1.02
7	20(−1)	12(+1)	12(+1)	0.99	0.94
8	30(+1)	12(+1)	12(+1)	1.08	1.12
9	16.59(‐α)	10(0)	10(0	0.96	0.97
10	33.41(+α)	10(0)	10(0)	1.29	1.24
11	25(0)	6.63(‐α)	10(0)	1.18	1.12
12	25(0)	13.36(+α)	10(0)	1.25	1.26
13	25(0)	10(0)	6.63(‐α)	1.34	1.34
14	25(0)	10(0)	13.36(+α)	1.05	1.02
15	25(0)	10(0)	10(0)	0.78	0.85
16	25(0)	10(0)	10(0)	0.77	0.85
17	25(0)	10(0)	10(0)	0.75	0.85
18	25(0)	10(0)	10(0)	0.79	0.85
19	25(0)	10(0)	10(0)	0.82	0.85
20	25(0)	10(0)	10(0)	0.89	0.85

### 2.6 Experimental design and methods of data analysis

Optimal fermentation time was determined by fermenting at previously optimized pH of 3.4, inoculum concentration of 10% (v/v), and 10% (v/v) Lantana camara fruit concentration for 2, 4, 6, 8, 10, and 12 days (Zenebe & Solomon, 2020). The fermentation process carried for two and four days has shown very low total titrable acidity (0.36 and 0.5%, w/v citric acid), below expected alcohol content (4 and 6%, v/v), as well as maximal residual sugar content (48 and 58 g/L equivalent to Dextrose glucose). The fermentation process conducted for eight, ten, and twelve days resulted wine with large total titrable acidity of 1.25, 1.43, and 1.55 (%, v/v citric acid), respectively, which are susceptible for microbial contamination. Hence, the fermentation process carried for six days produced wine with total titrable acidity of 1.05% (w/v citric acid) was selected as an optimal to control the fermentation time.

Experimental measurement uncertainties were determined to avoid cumulative error of the model developed which is reported in our pervious study Zenebe and Solomon (2020). Response surface method coupled with central composite rotatable design (CCRD) was employed to develop the experimental design as well as to investigate the statistical analysis of effects of fermentation temperature, yeast inoculum concentration, and concentration of Lantana camara fruit juice on total titratable acidity as shown in Table 1. The developed experimental design with 20 experimental runs shown in Table 1 of those 3 fermentation parameters have 6 axial points (±α), 6 center, and 8 factorial points. Polynomial function (Equation 1) was applied to determine critical points (maximum, minimum, or saddle) and for understanding impacts of independent fermentation variables on predicted total titratable acidity.

(1) $$Y=\beta_{0}+\sum_{i=1}^{3}\beta_{i}x_{i}+\sum_{i=1}^{3}\beta_{\mathrm{ii}}x_{i}^{2}+\sum_{i=1}^{3}\sum_{j=\left(i+1\right)}^{3}\beta_{\mathrm{ij}}x_{i}x_{j}$$

Where *Y* stands for total titratable acidity to be predicted; *i, j* represents linear, quadratic coefficients, correspondingly, $x_{i}$ and $x_{j}$ correspond to the three independent variables (fermentation temperature, inoculum concentration, and Lantana camara fruit concentration), and *β*_0_ (intercept), *β*_i_ (linear effects), *β*_ii_ (squared effects), and *β*_ij_(interaction terms) were for regression coefficients.

Statistical analysis software packages Design‐Expert version 10.0.3 (Stat‐Ease Inc., Minneapolis, MN, USA) was employed to obtain the model adequacies such as R^2^, adj‐R^2^, pre‐R^2^, Adeq. Precision, lack of fit, and C.V. which are shown in Table 3. Since the coefficients of determination (R^2^) increase as the number of input variables increases, it is not sufficient to criticize validity of the developing model. Hence, absolute average deviation (AAD) calculated using Equation 2 was additionally used to analyze the overall predictive capabilities of the developed model (Yolmeh & Jafari, 2017). Moreover, to show the difference between confirmatory experiment and predicted values as well as to investigate the adequacy of experimental data *t* test (α = 0.05) using Microsoft Excel 2013 version 15.0.4981.1001 (Microsoft Corporation, Redmond, Washington, USA) were employed.

(2) $$\text{AAD}=\left\{\left[\sum_{i=1}^{p}\left(\left|y_{i}\exp-y_{i}\text{cal}\right|/y_{i}\exp\right)\right]/p\right\}x100$$

Where *p, y_iexp_,* and *y_ical_* represent the number of experiment, experimental, and calculated responses, respectively.

### 2.7 Estimation of total titratable acidity (TTA)

Digital burette (Titrette, Brand Gmbh + Co KG, Wertheim, Germany) was used to determine the titration volume of wine samples (Zenebe & Kidu, 2019). As the procedure described by OIV (2016), each of 50 ml of filtered cactus pear and blended wine sample solution was taken into a 250 ml conical flask. First, carbon dioxide was removed by blowing air using vacuum pump into conical flask with continuous shaking for one to two minutes. Second, 10 ml of CO_2_ treated wine sample was poured into 30 ml of boiled distilled water and 1 ml of bromothymol blue solution contained conical flask. Next, sample solution was titrated with standardized 0.1 N NaOH to the bromothymol blue end point (blue‐green color). Bromothymol blue reference solution (color comparison) was prepared by mixing 1 ml of bromothymol blue solution, 25 ml of boiled distilled water, and 10 ml of CO_2_ treated wine sample in a 250 ml beaker. Then, sodium hydroxide solution (0.1 N), 5 ml of the pH 7 buffer solution was added until the color changes to blue‐green. Since the predominant acid in the cactus pear fruit and blend wine samples is citric acid, equivalent weight of citric acid was used to calculate total titratable acidity using the Equation 3.

(3) $$\text{TTA}\;\left(\%\frac{w}{v}\text{Predominant acid}\right)=\frac{NxV_{1}x\text{Eq}\text{.wt}.}{V_{2}x10}$$

Where *TTA* = total titratable acidity, *N* = normality of titrant (mEq/ml), *V_1_* = Volume of titrant (mL), *V_2_* = Volume of sample (mL) and Eq.wt. = Equivalent weight of tartaric acid or citric acid.

### 2.8 Determination of organic acids

Wine samples were first filtered by 0.45 µm Whatman nylon membrane filter (GE Healthcare co. USA). Then, a volume of 10 μl of the eluate was injected onto a C_18_ 70RBAX‐ODS column (250 x 4.6 mm and 5 µm particle size) (Agilent Technologies, USA) kept at 40 ᵒC of HPLC system equipped with a pump system (Model: series of 1260, Agilent Technologies, Palo Alto, CA‐USA) and a diode array detector (DAD) monitored at 210 nm ultraviolet–visible spectra to record all peaks (Kelebek, Selli, Canbas, & Cabaroglu, 2009). The separation was performed with isocratic elution (0.045 N H_2_SO_4_ and 6% acetonitrile (v/v)) During the analysis of organic acids in both wine samples, HPLC data were compared with external standards (0.002 gml^‐1^) of citric, L‐tartaric, L‐ascorbic, and oxalic acids developed as described in detail by Zenebe and Kidu (2019). Organic acid concentration in each wine sample was estimated using the relative peak area covered in the chromatogram and calculated using Equation 4.

(4) $$\text{Organic acid concentration}\left(\frac{\text{mg}}{\text{mL}}\right)=\frac{\text{Peak area of sample}}{\text{Peak area of standard}}\;\text{x Concentration of standard}$$

## 3 RESULTS AND DISCUSSION

### 3.1 Fitting the response surface model

The response surface methodology employed optimization of the effect of fermentation temperature; inoculum and Lantana camara fruit concentration on total titratable acidity of the blended wine was investigated. The total titratable acidity response was developed using the experimental design shown in Table 1. Polynomial equation developed for predicting total titratable acidity of the produced wine (Equation 5) is function of fermentation temperature: inoculum and Lantana camara fruit concentration. The calculated predicted values of Equation 5 are presented in Table 1. Using Design Expert software package, optimum levels of fermentation temperature, inoculum concentration, and Lantana camara fruit juice concentration were predicted by applying regression analysis on this equation.

(5) $$\text{Total Titrable Acidity}\left(\text{TTA}\%\frac{w}{v}\right)=8.24-0.21x\text{Temp}-0.7x\;\text{InC}.-0.27x\text{LFC}+0.01x\text{Temp}x\text{InC}.-0.009x\text{Temp}x\text{LFC}-0.022x\text{InC}.x\text{LFC}+0.0043x{\text{Temp}}^{2}+0.035x\text{InC}.^{2}+0.033x{\text{LFC}}^{2}$$

Temp, InC., and LFC are represented for fermentation temperature, inoculum concentration, and Lantana camara fruit juice concentration.

Adequacy of the developed model was checked using absolute average deviation (AAD), pre‐R^2^, adequacy of precision, PRESS, and adj‐R^2^ as shown in Table 3. As shown in Table 2, *F‐*value (27.95) of the total titratable acidity predicting equation implies the developed model is very significant (*p* < .0001) and there is only 0.01% chance that the models *F*‐value this large could occur due to noise. This indicates the predicting model equation is sufficiently accurate to predict the total titratable acidity quality of the fermentation process. High probability value of the model experimental data represents insignificant lack of fit of the model. Hence, the developed predictive model is sufficiently accurate for predicting the relevant response of the fermentation process.

**Table 2:** ANOVA evaluation of linear, interaction, and quadratic terms for total titratable acidity variables and coefficients of the model prediction

Source	Estimated Coefficient	Sum of Squares	*df*	Mean Square	*F*‐ value	*p*‐value	
Model	0.80	1.08	9	0.12	27.95	<.0001	significant
*A‐Temperature*	0.077	0.081	1	0.081	19.00	.0014	
*B‐Inoculum Concentration*	0.055	0.042	1	0.042	9.80	.0107	
*C‐Concentration of L.Fruit*	−0.11	0.16	1	0.16	37.79	.0001	
*AB*	0.10	0.080	1	0.080	18.65	.0015	
*AC*	−0.090	0.065	1	0.065	15.11	.0030	
*BC*	−0.087	0.061	1	0.061	14.28	.0036	
*A^2^*	0.11	0.17	1	0.17	39.03	<.0001	
*B^2^*	0.14	0.28	1	0.28	65.48	<.0001	
*C^2^*	0.13	0.25	1	0.25	59.01	<.0001	
Residual		0.043	10	0.0043			
*Lack of Fit*		0.030	5	0.0061	2.46	.1730	*not significant*
*Pure Error*		0.012	5	0.0025			
Cor Total		1.12	19				

As revealed in Table 3, the model has coefficient of determination (R^2^) 96.18% which implies the model is well fitted. Suitability of the fitting empirical model to the actual data is indicated when R^2^ approaches to unity where the current R^2^ is 0.9618 (Peng et al., 2015). But, R^2^ index alone cannot demonstrate the accuracy of the model since increasing more input variable to the model will increase R^2^ exclusively expressed the statistical significance of the additional variable. Therefore, analysis of statistical dispersion or variability from a central point should be considered by calculating absolute average deviation (AAD) indicated in Equation 2. The calculated percentile of absolute average deviation (%AAD) between the estimated and observed data is shown in Table 3. The value must be low to depict correct behavior of the fitted model on the fermentation process which can be successfully used for the optimization of the wine fermentation processes (Yolmeh & Jafari, 2017). As it is presented in Table 3, the estimated and observed data of the fermentation optimization process are varied by 2.06% which relates to the coefficient of determination (R^2^) of the developed model.

**Table 3:** Experimental data analysis of the total titratable acidity model

Statistical parameter	Statistical value	Statistical parameter	Statistical value
Std. Dev.	0.065	Adjusted R^2^	0.9274
Mean	1.06	Predicted R^2^	0.7693
C.V. (%)	6.18	Adequacy of Precision	19.415
PRESS	0.26	AAD (%)	2.06
R^2^	0.9618		

Note

Where Std.Dev., C.V., PRESS and AAD stands for standard deviation, coefficient of variation, predicted regression error sum of square and absolute average deviation, respectively.

### 3.2 Evaluating fermentation variables effect on total titratable acidity.

More than one variable at a time analysis of variance (ANOVA) was used to analyze the effect of each input factor and their interaction on total titratable acidity of the blended wine which is presented in Table 2. Regression coefficients of the developed quadratic polynomial model and corresponding coefficient values of determination (R^2^) are also shown in Table 2. From the ANOVA (Table 2), it was observed that all the linear, interaction, and quadratic coefficients of the developed model have significant (*p* ≤ .01) effect on the total titratable acidity of the final wine. Hence, all the regression coefficients were used to develop the total titratable acidity predictive model (Equation 5).

#### 3.2.1 Effect of fermentation temperature on total titratable acidity of the blended fruit wine

**Figure 1:**
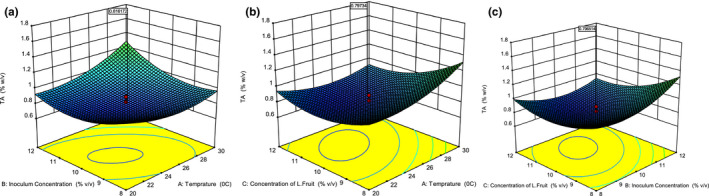
Response surface and contour plots for the effect of inoculum concentration (a, Lantana camara fruit concentration = 10% v/v) and temperature; Lantana camara fruit concentration (b, inoculum concentration = 10% v/v) and temperature; Lantana camara fruit concentration (c, temperature = 25°C) and inoculum concentration on the total titrable acidity of the blended wine

As it can be seen in Table 2, the fermentation temperature significantly (*p* ≤ .001) affected the total titratable acidity of the produced blended wine linearly. Similarly, interaction as well as quadratic effect of fermentation temperature significantly (*p* ≤ .001) influenced total titratable acidity of the final wine. The interaction effect of fermentation temperature with inoculum concentration is significant in which only 0.15% of its interaction is not explained. Fruit fermentation process facilitates extensive mycelial growth when temperature increases up to the maximum tolerable limit of the yeast strain used. The total titratable acidity produced at 25°C is maximum (0.81%, w/v) which is shown in Figure 1a. Moreover, the interaction effect of fermentation temperature with Lantana camara fruit juice concentration on total titratable acidity of the final wine was significant (*p* < .01). As it is depicted in Figure 1c, as fermentation temperature and concentration of Lantana camara fruit juice increase total titrable acidity enhanced exponentially. A study conducted by Ogodo et al. (2015) on fermentation of mixed fruits of pawpaw, banana, and watermelon to produce blended fruit wine reported suitable temperature ranged from 27 to 28°C. Above this optimum temperature range, large mass of sugars oxidized to CO_2_ which slows down the production yield of citric acid but enhances oxalic acid and gluconic acids production yield (Ambati & Ayyanna, 2001). As fermentation temperature increased the amount of succinic and acetic acid significantly enhanced during strains of *S. cerevisiae* fermentation of grape must. Moreover, the influence of yeast type and fermentation temperature on acetic acid and citric acid concentrations significantly varied at 18°C and 21°C (Chidi et al., 2018).

#### 3.2.2 Effect of inoculum concentration on total titratable acidity of the blended fruit wine

Linear and interaction effects of the inoculum concentration on the total titratable acidity of the final wine are significant (*p* ≤ .01) which is presented in Table 2. Similarly, total titratable acidity of the final wine is significantly (*p < *.0001) affected by quadratic effect of the inoculum concentration. Inoculum concentration in fermenting substrate plays significant role by consuming and converting the amount of nutrients during glycolysis inside the mitochondria of the yeasts’ cell (Vilela, 2019). Alcohols and organic acids are produced during the fermentation process of fruit juice substrate in the presence of *Saccharomyces cerevisiae*. During the fermentation process, yeasts convert sugars into alcohol and important organic acids, such as succinic, pyruvic, lactic, and acetic acid, which are responsible to change total titratable acidity of the final wine. Similarly, yeast strains intrinsically grow at different temperature‐dependent fermentation process that tends to produce succinic acid and acetic acids which are not studied in the current study (Chidi et al., 2018). But, anthocyanin compounds are adsorbed during the fermentation process by cell walls of the yeast strains which determines tartaric acid and color property of the final wine (Ogodo et al., 2015). As it can be seen in Figure 1b, increasing inoculum and Lantana camara fruit juice concentration increased total titratable acidity of the produced blended wine. This could be due to some new organic acids such as acetic acid could be produced during the fermentation process (due to microbiological activities), and some additional organic acids could be incorporated from the Lantana camara fruit juice added. Zenebe &Kidu (2019) reported that the dominant organic acid present in Lantana camara fruit is tartaric acid which could participate on the total titratable acidity enlargement.

#### 3.2.3 Effect of Lantana camara fruit juice concentration on total titratable acidity of the blended fruit wine

The Lantana camara blended with cactus pear fruit has significantly (*p < *.001) influenced total titratable acidity of the final wine (Table 2). Moreover, the Lantana camara fruit juice added interactively with fermentation temperature and inoculum concentration showed significant (*p* ≤ .01) effect on total titratable acidity content of the produced wine. Quadratic terms of the blended Lantana camara fruit juice significantly (*p < *.0001) influenced the fermentation process which impacted total titratable acidity of the final wine. As it can be seen in Figure 1b and 1c, blending Lantana camara fruit juice slightly enhanced total titratable acidity of the produced wine. Similar situation was observed during the production of mixed fruits wine (pawpaw, watermelon, and pawpaw fruits wine) (Ogodo et al., 2015). Vitamins and nicotinic acid present in fruits are limiting factor for lactic acid production during fermentation since these are commonly required by yeast as organic cofactors for the enzymatic complexes reactions (Chidi et al., 2018). Hence, the total titratable acidity property of the produced wine was dependent on the presence of the vitamins and nicotinic acids.

### 3.3 Optimization of significant factors

Response surface methodology coupled with central composite rotatable design (CCRD) enables to develop optimum fruit juice fermentation conditions to produce quality fruit wine (Peng et al., 2015). As shown in Figure 1, the optimum fermentation temperature and inoculum concentration at constant Lantana camara fruit juice concentration (10%, v/v) to produce 0.81% (w/v equivalent to citric acid) total titratable acidity of the final wine are 25°C and 10%, v/v, respectively. Furthermore, the optimum inoculum and Lantana camara fruit juice concentration at constant temperature (25°C) to produce total titratable acidity of 0.8 ± 0.058% (w/v equivalent to citric acid) are both 10%, v/v.

The ellipses or circles contour plots displayed in Figure 1 depict the center of points of minimum total titratable acidity occur. These contours (or surfaces) represent contours of the estimated total titratable acidity of the final wine and the general nature of the fermentation process arose as a result of the fitted model. The minimum points (stationary points) of the developed second order equation (Equation 5) are the point where the first derivative of the function equals to zero (Baş & Boyacı, 2007).

$$\frac{\partial \text{TTA}}{\partial \text{Temp}}=\frac{\partial }{\partial \text{Temp}}\left(-0.21\text{Temp}+0.01\text{Temp}x\text{InC}.-0.009\text{Temp}x\text{LFC}+0.0043{\text{Temp}}^{2}\right)=0$$

=-0.21+0.01InC.-0.009LFC+0.0086Temp=0

$$\frac{\partial \text{TTA}}{\partial \text{InC}.}=\frac{\partial }{\partial \text{InC}.}\left(-0.7\text{InC}.+0.01\text{Temp}x\text{InC}.-0.022\text{InC}.x\text{LFC}+0.035\text{InC}.^{2}\right)=0$$

$$\frac{\partial \text{TTA}}{\partial \text{LFC}}=\frac{\partial }{\partial \text{LFC}}(-0.27\text{LFC}-0.009\text{Temp}x\text{LFC}-0.022\text{InC}.x\text{LFC}+0.033{\text{LFC}}^{2}=0$$

=-0.27-0.009Temp-0.022InC.+0.066LFC=0

Where TTA, Temp, InC., and LFC represent for total titrable acidity, fermentation temperature, inoculum concentration, and Lantana camara fruit juice concentration.

Solving these equations (i, ii, and iii) using excel solver, the optimum fermentation temperature, and inoculum and Lantana camara concentration to produce 0.8% (w/v equivalent to citric acid) total titratable acidity of the blended wine are 24.06°C, 9.92% (v/v), and 10.68% (v/v), respectively. Similar optimum predictive values of the fermenting variable were also determined using the statistical analysis software packages Design‐Expert version 10.0.3 as shown in Table 4.

**Table 4:** Confirmation report to validate the combination of fermentation parameters on the total titratable acidity predictive model

Factor code	Factor name	Factor optimum Level	Factor low level	Factor high level	Std. Dev.	Factor coding	
A	Temperature	23.95	16.6	33.4	0.000	Actual	
B	Inoculum Concentration	9.96	6.6	13.4	0.000	Actual	
C	Concentsration of L.Fruit	10.67	8.6	13.4	0.000	Actual	
**Response**	**Response predicted mean**	**Response predicted median**	**Response Std Dev**	**Response S.E. Pred**	**Response 95% PI low**	**Response measured data mean**	**Response 95% PI high**
Total titratable acidity	0.774284	0.774284	0.0654905	0.042	0.68	0.83	0.87

Note.

The confirmation report is with two‐sided confidence (95%) for three confirmation.experiments; PI, predictive interval; Std Dev, standard deviation; S.E. Pred, predicted sum of errors.

**Figure 2:**
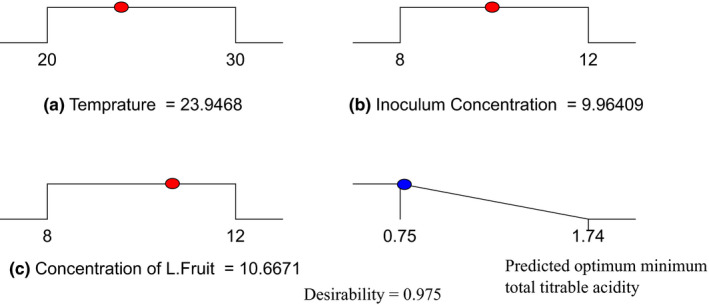
Desirability ramp for numerical optimization of each input factor and predicted optimum minimum total titrable acidity of the blended wine

Desirability function approach was employed to get the combined optimum fermentation parameters (Bezerra, Santelli, Oliveira, Villar, & Escaleira, 2008). Design‐Expert software based one‐sided transformation was applied to get optimum minimum total titrable acidity of the produced wine when the desirability function approaches to unity. As it was depicted in Figure 2, the optimized fermentation temperature, inoculum concentration, and Lantana camara fruit concentration at 0.975 desirability function were 24°C, 10% (v/v), and 10.7% (v/v) to produce wine with total titratable acidity of 0.774 ± 0.065% (w/v equivalent to citric acid). Figure 2 was developed from optimum points via numerical optimization using this software to show desirability for each predictive factor and total titratable acidity vales of the produced wine.

### 3.4 Validation and confirmation of the developed total titrable acid predictive model

Adequacy of the developed total titratable acidity predictive model for the blended wine was verified, and the analyzed of results are shown in Table 3. As it can be seen in Table 3, the standard deviation, coefficient of determination R^2^, Adjusted R^2^, Predicted R^2,^ and %AAD are 0.065, 0.96, 0.927, 0.77, and 2.06, respectively. The analyzed result implies that the studied fermentation input factors had significant effects on the total titratable acidity of the final wine, and the relationship between factor variability and the total titratable acidity was real and reliable. Moreover, adequacy of precision was measured as 19.42 (Table 3) indicating the developed model had an adequate signal to predict the total titratable acidity of the blended wine in which a ratio greater than 4 is desirable. The Predicted R^2^ of 0.7693 is in reasonable agreement with the Adjusted R^2^of 0.9274; that is, the difference is less than 0.2 (Montgomery, 2017). As it is also described in Section 3.4, the predictive optimum fermentation temperature, and inoculum and Lantana camara fruit concentration determined using the statistical analysis software packages Design‐Expert are shown in Table 4. At these optimum fermentation parameters, the predictive total titratable acidity of the blended wine is about 0.8% (w/v equivalent to tartaric acid) (Table 4).

Three confirmatory experiments were conducted to validate the combination of these three fermentation parameters. At the optimum fermentation temperature of 24°C, inoculum concentration 10% (v/v), and Lantana camara fruit juice concentration of 10.7% (v/v), the produced blended wine has total titratable acidity of 0.83 ± 0.058% (w/v equivalent to citric acid). This result perfectly matched with the predicted value of 0.8 ± 0.065% (w/v equivalent to citric acid) at *t* test (t_0.05,2_) without significant difference as shown in Table 4.

### 3.5 Total titratable acidity analysis of wines produced from cactus pear and blended with Lantana camara fruit

**Figure 3:**
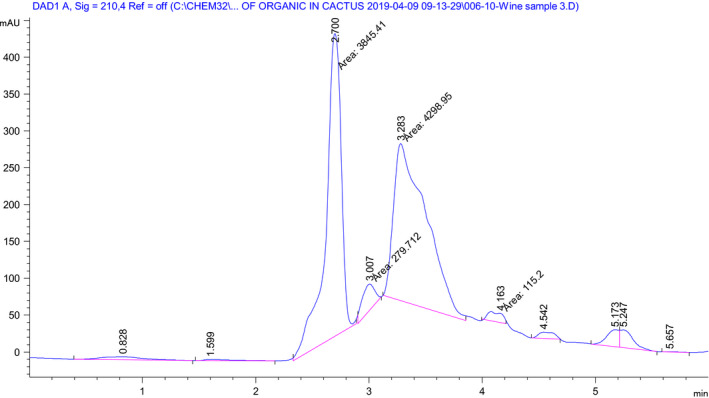
High performance liquid chromatography (HPLC) chromatogram for organic acids of the cactus pear fruit wine at 210 nm (peak area: citric acid = 4,208.95; L‐tartaric acid = 3,845.41; L‐ascorbic acid = 279.712; oxalic acid = not detected and others are unknown)

Cactus pear fruit wine was produced at previously optimized fermentation process (at pH of 3.4 and 16% inoculum concentration and at temperature of 30°C) to study the total titratable acidity difference with the wine produced by blending cactus pear and Lantana camara fruits optimized in the current study of fermentation temperature 24°C, inoculum concentration 10% (v/v), and Lantana camara fruit concentration of 10.67% (v/v) (Zenebe et al., 2018). Based on the organic acid analysis depicted in Figure 3 and Table 5, the dominant organic acid present in the cactus pear fruit wine alone is citric acid. Thus, total titratable acidity analysis was 1.06 ± 0.27% (w/v) considering equivalent weight of citric acid. This elevated total titrable acidity indicates the fermentation process needs further malolactic fermentation to reduce the acidity. Cactus pear fruit juice dominantly contained tartaric acid (Zenebe & Kidu, 2019). Tartaric acid was also used during the fermenting substrate pH adjustment; the second abundant organic acid in the produced wine is tartaric acid. As it was reported in our previous study, the dominant acid present in cactus pear and Lantana camara fruit juices is also tartaric acid. Even though tartaric acid was used for the fermentation substrate acidification, the main acid present in the blended fruit wine is citric acid which could be due to the difference fermentation process applied. The total titratable acidity of the wine produced by blending these fruits is reported as equivalent to citric acid (Zenebe & Kidu, 2019). As it can be seen in Table 5, total titratable acidity of the cactus pear fruit wine is 1.06% (w/v citric acid) which is larger than (0.83%, v/v citric acid) the wine produced by blending cactus pear and Lantana camara fruits. But lower than the total titratable acidity reported by Kelebek et al. (2009) for orange fruit wine (6.3 g/L), Panda, Sahu, Behera, and Ray (2014b) for sapota fruit wine (1.29 g/100 ml). During the Kiwifruit wine fermentation, three organic acids (citric, malic, and tartaric acid) contributed to the significant decline in total titratable acidity from 74.13% to 69.49% (Zhong et al., 2020). During yeast inoculation due to high initial sugar concentrations, alcoholic fermentation starts at hyperosmotic stress conditions. At this situation, wine yeasts could produce acetic acid to response the hyperosmotic stress which increases total titratable acidity in the produced wine (Chidi et al., 2018). Commercial yeasts are less resistant to high sugar content and temperature and incapable to degrade citric, malic, and tartaric acid present in fermenting fruit substrate. Hence, fermented fruit wine using these yeasts tends to contain significant total titratable acidity (Zhong et al., 2020). Similarly, variations of fermentation temperature and pH during fruit wine production could significantly affect the evolving of succinic and pyruvic acids which influences the total titratable acidity of the final wine (Chidi, Rossouw, Buica, & Bauer, 2015).

**Table 5:** Total titratable acidity and organic acids of wines produced from cactus pear fruit and by blending with Lantana camara fruit

Parameter	Cactus pear fruit wine Mean, Std Dev	Blended fruit Wine Mean, Std Dev
Total titratable acidity (% w/v Citric acid)	1.06 ± 0.27	0.83 ± 0.058
Organic acids (mg/mL)		
Citric acid	7.09 ± 0.07	4.74 ± 0.07
L‐tartaric acid	4.77 ± 0.25	1.06 ± 0.36
L‐ascorbic acid	0.05 ± 0.09	0.12 ± 0.07
Oxalic acid	ND	ND
Total organic acid content	11.91 ± 0.37	5.92 ± 0.5

Note

ND, Not detected; Std Dev, standard deviation.

The total titratable acidity of the wine produced from cactus pear and Lantana camara fruits blend is consistent with the study reported by Ogodo et al. (2015) for mixed fruit wine (0.35 to 0.88%), Reddy and Reddy (2005) for mango fruit wine (from 0.6% to 0.8%, v/v), and Joshi, Kumar, and Kumar (2012) for mandarin and kinnow fruit wines (0.86%). But the current result is higher than the wine produced from watermelon juice and ginger extract blended (from 0.04% to 0.10%) reported by Yusufu et al. (2018), and Panda, Sahu, Behera, and Ray (2014a) for bael wine 0.15 g/100ml. In the study reported by Zhong et al. (2020), total titratable acidity of Kiwifruit wine was significantly decreased after fermentation due to citric, malic, and tartaric acid reduced from 12.3, 3.09, and 0.61 g/L to 11.00, 2.02, and 0.41 g/L, respectively. In this study, using pure‐cultured yeasts are more recommended than commercial yeasts which can tolerate high sugar content and temperature during fermentation process to enhance organic acid degradation (decrease total titratable acidity).

Important organic acids, such as succinic, pyruvic, lactic, and acetic acid, are produced during fruit juice fermentation process in the presence of yeast and bacteria (Chidi et al., 2018). Thus, total titratable acidity in fruit wines is related to the total number of protons of all undissociated organic acids present in the substrate. Since higher quantity of organic acids such as acetic acid in the wine is related to poor fruit wine quality, acetic acid content of fruit wine should be below 1.2 g/L or according to the legal limits countries (Dias et al., 2017). But, ice wines and botrytized wines can reach a maximum acetic acid concentration of 2.1 g/L. Total titratable acidity of both fruit wines produced in the current study is below this limit (Table 5). During the production of wine from sapota fruit, total titratable acidity increased from 0.82 (g tartaric acid/100 ml) in must to 1.29 (g tartaric acid/100 ml) in wine that is reported by Panda et al. (2014b). In this study, accumulation of organic acids such as lactic and ascorbic acids during fermentation process minimizes the influence of spoilage bacteria which affects the wine to have acidic characteristic.

### 3.6 Organic acid analysis of wines produced from cactus pear and blended with Lantana camara fruit

The HPLC analysis of organic acids in cactus pear fruit wine alone revealed the presence of citric acid, L‐tartaric acid, L‐ascorbic acid, and other more unidentified organic acids (Figure 3). Even though oxalic acid was identified in a cactus pear fruit wine reported by Navarrete‐Bolaños et al. (2013) and expected to get in the current study, it was not detected in both wines. This can be due to the difference in origin of the cactus pear fruit as well as the fermentation process adopted. The predominant organic acid present in cactus pear fruit is L‐tartaric acid reported by Zenebe &Kidu (2019). Besides, tartaric acid was used for acidification (pH adjustment) during the cactus pear fruit juice substrate preparation. Tartaric acid is more resistant to microbial breakdown during fermentation process which achieves less off‐flavor compared to citric acid and L‐malic acid (Volschenk, Van Vuuren & Viljoen‐Bloom, 2006). These could the main reason to raise the tartaric acid concentration in the produce wine. As it can be seen in Figure 3, major organic acid present in the cactus pear fruit wine is citric acid. The second and third organic acids identified using the HPLC analysis are L‐tartaric acid and L‐ascorbic acids, respectively. Concentrations of each identified organic acids were calculated using Equation 4, and the total organic acid was reported in Table 5. Tartaric acid was also identified in a young cactus pear fruit wine produced from *Opuntia streptacantha* variety originated from Mexico using HPLC method (Navarrete‐Bolaños et al., 2013). Wine produced from gabiroba fruit contained organic acids such as citric acid (3.13 ± 0.25 g/L), tartaric acid (1.02 ± 0.11 g/L), and oxalic acid (0.26 ± 0.06 g/L) determined using HPLC (Duarte, Dias, de Melo Pereira, Gervásio, & Schwan, 2009). In this study, the oxalic acid was identified in the final wine but it was deficient in the fermenting substrate. From the study reported by Jitjaroen, Bouphun, and Panjai (2013), Mao wine produced using malolactic fermentations significantly reduced citric acid whereas the concentration of tartaric acid increased at the end of the fermentation. This indicates yeast strains perform differently on the type of organic acid present on the fermenting substrate.

**Figure 4:**
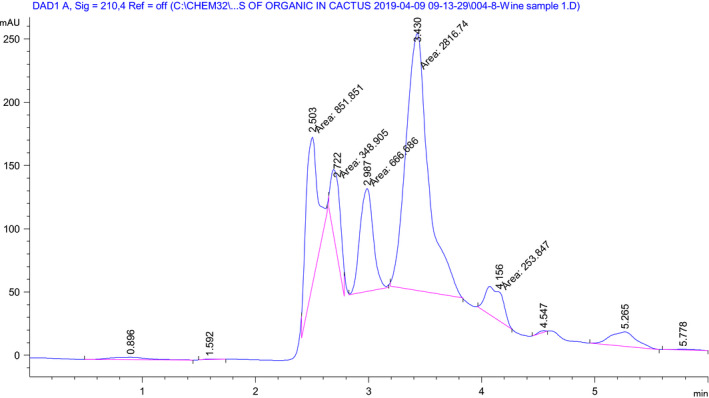
High performance liquid chromatography (HPLC) chromatogram for organic acids of the cactus pear and Lantana camara fruits blended wine at 210 nm (peak area: citric acid = 2,816.74; L‐tartaric acid = 851.851; L‐ascorbic acid = 666.686; oxalic acid = not detected and others are unknown)

Figures 3 and 4 depict the HPLC chromatogram peak area difference as well as the additional organic acids produced in the cactus pear fruit alone and blended with Lantana camara fruit wine fermentation process correspondingly. Based on the HPLC measurements of the blended fruit wine, the predominant organic acid identified was citric acid (about 80.07% of the total organic acid identified) as shown in Table 5. The next abundant organic acid was L‐tartaric acid (1.06 ± 0.36 mg/ml), and the least predominant acid was L‐ascorbic acid (0.12 ± 0.07 mg/ml) which was also depicted in Figure 4. The blended Lantana camara fruit changed the fermentation condition of the cactus pear fruit juice substrate. As it can be seen in Table 5, concentration of citric acid decreased (4.74 ± 0.07 mg/ml) due to the addition of Lantana camara fruit juice compared to wine produced from cactus pear fruit (7.09 ± 0.07 mg/ml) only. Even though there is lack of evidence that the yeast strain *Saccharomyces cerevisiae* can effectively transport or degrade tartaric acid, other unknown bio‐chemical transformations occurred during fermentation could lower the acid at the end of fermentation. The purple color present in Lantana camara fruit could precipitate organic acids, especially the tartaric acid (copigmentation with anthocyanins) (Chidi et al., 2015; Volschenk et al., 2006). Cactus pear (83.6%) and Lantana camara (83.8%) fruit juices contain dominantly tartaric acid of the identified organic acid (Zenebe & Kidu, 2019). Yeasts and other organisms use citric acid as important intermediate central carbon metabolism in the tricarboxylic acid cycle (TCA cycle) (Vilela, 2019). During the gabiroba fruit fermentation process for wine production, citric acid, tartaric acid, and oxalic acid increased after fermentation from 1.72, 0.78, and 0.0 g/L to 3.13, 1.02, and 0.26 g/L, respectively (Duarte et al., 2009). In general, the produced wines have shown difference in total titrable acidity and composition of organic acids.

## 4 CONCLUSION

The developed mathematical model to predict total titrable acidity of the blended fruit wine is sufficiently accurate. The cactus pear and Lantana camara blended fruit wine produced using response surface optimization has shown lower total titrable acidity (0.83 ± 0.27 w/v citric acid) compared to the wine produced from cactus pear fruit alone (1.06 ± 0.27 w/v citric acid). In both types of fruit wines, considerable organic acids of citric, L‐tartaric, and L‐ascorbic acid were identified in addition to other nonidentified acids. In both fruit wines, the predominant organic acid was citric acid. The total organic acid concentration produced in cactus pear fruit was larger (11.91 ± 0.37) than the blended fruit wine (5.92 ± 0.5). In general, the analyzed total titrable acidity and organic acids can vary organoleptic properties, microbiological and biochemical stability of the produced wines. Further studies considering optimization of volatile acids and other organoleptic determining factors are vital to have full properties of the blended fruit wine. Application of integrated yeast (*Saccharomyces cerevisiae*) strain and malolactic fermentation process to reduce the elevated citric acid concentration cactus pear fruit needs further investigation.

## CONFLICT OF INTEREST

The author declares that there is no conflict of interest that could be perceived as prejudicing the impartiality of the research reported.

## ETHICAL APPROVAL

This study does not involve any human or animal testing.

## References

[fsn31745-bib-0001] Al‐Juhaimi, F. , & Özcan, M. M. (2013). Determination of some mineral contents of prickly pear (Opuntia ficus‐indica L.) seed flours. Environmental Monitoring and Assessment, 185(5), 3659–3663. 10.1007/s10661-012-2817-4 22886626

[fsn31745-bib-0002] Ambati, P. , & Ayyanna, C. (2001). Optimizing medium constituents and fermentation conditions for citric acid production from palmyra jaggery using response surface method. World Journal of Microbiology and Biotechnology, 17(4), 331–335.

[fsn31745-bib-0003] Baş, D. , & Boyacı, I. H. (2007). Modeling and optimization I: Usability of response surface methodology. Journal of Food Engineering, 78(3), 836–845. 10.1016/j.jfoodeng.2005.11.024

[fsn31745-bib-0004] Belviranlı, B. , Al‐Juhaimi, F. , Özcan, M. M. , Ghafoor, K. , Babiker, E. E. , & Alsawmahi, O. N. (2019). Effect of location on some physico‐chemical properties of prickly pear (Opuntia ficus‐indica L.) fruit and seeds. Journal of Food Processing and Preservation, 43(3), e13896 10.1111/jfpp.13896

[fsn31745-bib-0005] Bezerra, M. A. , Santelli, R. E. , Oliveira, E. P. , Villar, L. S. , & Escaleira, L. A. (2008). Response surface methodology (RSM) as a tool for optimization in analytical chemistry. Talanta, 76(5), 965–977. 10.1016/j.talanta.2008.05.019 18761143

[fsn31745-bib-0006] Carstairs, S. D. , Luk, J. Y. , Tomaszewski, C. A. , & Cantrell, F. L. (2010). Ingestion of Lantana camara is not associated with significant effects in children. Pediatrics, 126(6), e1585–e1588. 10.1542/peds.2010-1669 21041281

[fsn31745-bib-0007] Chidi, B. S. , Bauer, F. F. , & Rossouw, D. (2018). Organic acid metabolism and the impact of fermentation practices on wine acidity: A review. South African Journal of Enology and Viticulture, 39(2), 1–15. 10.21548/39-2-3164

[fsn31745-bib-0008] Chidi, B. S. , Rossouw, D. , Buica, A. S. , & Bauer, F. F. (2015). Determining the impact of industrial wine yeast strains on organic acid production under white and red wine‐like fermentation conditions. South African Journal of Enology and Viticulture, 36(3), 316–327. 10.21548/36-3-965

[fsn31745-bib-0009] Coelho, E. , Vilanova, M. , Genisheva, Z. , Oliveira, J. M. , Teixeira, J. A. , & Domingues, L. (2015). Systematic approach for the development of fruit wines from industrially processed fruit concentrates, including optimization of fermentation parameters, chemical characterization and sensory evaluation. LWT ‐ Food Science and Technology, 62(2), 1043–1052. 10.1016/j.lwt.2015.02.020

[fsn31745-bib-0010] Darias‐Martín, J. , Socas‐Hernández, A. , Díaz‐Romero, C. , & Díaz‐Díaz, E. (2003). Comparative study of methods for determination of titrable acidity in wine. Journal of Food Composition and Analysis, 16(5), 555–562. 10.1016/S0889-1575(03)00032-2

[fsn31745-bib-0011] Dias, D. R. , Duarte, W. F. , & Schwan, R. F. (2017). Methods of evaluation of fruit wines. Science and Technology of Fruit Wine Production. 10.1016/B978-0-12-800850-8.00005-3

[fsn31745-bib-0012] Duarte, W. F. , Dias, D. R. , de Melo Pereira, G. V. , Gervásio, I. M. , & Schwan, R. F. (2009). Indigenous and inoculated yeast fermentation of gabiroba (Campomanesia pubescens) pulp for fruit wine production. Journal of Industrial Microbiology & Biotechnology, 36(4), 557–569. 10.1007/s10295-009-0526-y 19190949

[fsn31745-bib-0013] Jitjaroen, W. , Bouphun, T. , & Panjai, L. (2013). The potential of malolactic fermentation on organic acids degradation in Mao (Antidesma Thwaitesanum Müell.) wine production. International Journal of Bioscience, Biochemistry and Bioinformatics, 3(4), 368–371. 10.7763/IJBBB.2013.V3.234

[fsn31745-bib-0014] Joshi, V. , Kumar, V. , & Kumar, A. (2012). Physico‐chemical and sensory evaluation of wines from different citrus fruits of Himachal Pradesh. International Journal of Food and Fermentation Technology, 2(2), 145.

[fsn31745-bib-0015] Kelebek, H. , Selli, S. , Canbas, A. , & Cabaroglu, T. (2009). HPLC determination of organic acids, sugars, phenolic compositions and antioxidant capacity of orange juice and orange wine made from a Turkish cv. Kozan. Microchemical Journal, 91(2), 187–192. 10.1016/j.microc.2008.10.008

[fsn31745-bib-0016] Lee, J.‐H. , Kang, T. H. , Um, B. H. , Sohn, E.‐H. , Han, W.‐C. , Ji, S.‐H. , & Jang, K.‐H. (2013). Evaluation of physicochemical properties and fermenting qualities of apple wines added with medicinal herbs. Food Science and Biotechnology, 22(4), 1039–1046. 10.1007/s10068-013-0181-y

[fsn31745-bib-0017] Matthäus, B. , & Özcan, M. M. (2011). Habitat effects on yield, fatty acid composition and tocopherol contents of prickly pear (Opuntia ficus‐indica L.) seed oils. Scientia Horticulturae, 131, 95–98. 10.1016/j.scienta.2011.09.027

[fsn31745-bib-0018] Montgomery, D. C. (2017). Introduction to Response Surface Methodology Design and analysis of experiments, Eighth Edition(478–555). United States: John wiley & sons.

[fsn31745-bib-0019] Navarrete‐Bolaños, J. , Fato‐Aldeco, E. , Gutiérrez‐Moreno, K. , Botello‐Álvarez, J. , Jiménez‐Islas, H. , & Rico‐Martínez, R. (2013). A strategy to design efficient fermentation processes for traditional beverages production: Prickly pear wine. Journal of Food Science, 78(10), M1560–M1568. 10.1111/1750-3841.12237 24032574

[fsn31745-bib-0020] Nikhanj, P. , & Kocher, G. (2018). Statistical optimization of ethanol fermentation parameters for processing local grape cultivars to wines. Journal of Food Processing and Preservation, 42(1), e13319 10.1111/jfpp.13319

[fsn31745-bib-0021] Ogodo, A. C. , Ugbogu, O. C. , Ugbogu, A. E. , & Ezeonu, C. S. (2015). Production of mixed fruit (pawpaw, banana and watermelon) wine using Saccharomyces cerevisiae isolated from palm wine. SpringerPlus, 4(1), 683 10.1186/s40064-015-1475-8 26576326 PMC4639538

[fsn31745-bib-0022] OIV (2016). Compendium of international methods of wine and must analysis, Vol. 1 (pp. 331–335). Paris.

[fsn31745-bib-0023] Özcan, M. M. , & Al Juhaimi, F. Y. (2011). Nutritive value and chemical composition of prickly pear seeds (Opuntia ficus indica L.) growing in Turkey. International Journal of Food Sciences and Nutrition, 62(5), 533–536. 10.3109/09637486.2011.552569 21391790

[fsn31745-bib-0024] Panda, S. K. , Sahu, U. C. , Behera, S. K. , & Ray, R. C. (2014a). Bio‐processing of bael [Aegle marmelos L.] fruits into wine with antioxidants. Food Bioscience, 5, 34–41. 10.1016/j.fbio.2013.10.005

[fsn31745-bib-0025] Panda, S. K. , Sahu, U. C. , Behera, S. K. , & Ray, R. C. (2014b). Fermentation of sapota (Achras sapota Linn.) fruits to functional wine. Nutrafoods, 13(4), 179–186. 10.1007/s13749-014-0034-1

[fsn31745-bib-0026] Peng, B. , Lei, Y. , Zhao, H. , & Cui, L. (2015). Response surface methodology for optimization of fermentation process parameters for improving apple wine quality. Journal of Food Science and Technology, 52(11), 7513–7518. 10.1007/s13197-015-1872-6

[fsn31745-bib-0027] Reddy, L. V. A. , & Reddy, O. V. S. (2005). Production and characterization of wine from Mango Fruit (Mangifera indica L). World Journal of Microbiology and Biotechnology, 21(8), 1345–1350. 10.1007/s11274-005-4416-9

[fsn31745-bib-0028] Sousa, E. O. , Rocha, J. B. , Barros, L. M. , Barros, A. R. , & Costa, J. G. (2013). Phytochemical characterization and in vitro antioxidant properties of Lantana camara L. and Lantana montevidensis Briq. Industrial Crops and Products, 43, 517–522. 10.1016/j.indcrop.2012.07.058

[fsn31745-bib-0029] Tadesse, E. , Engidawork, E. , Nedi, T. , & Mengistu, G. (2017). Evaluation of the anti‐diarrheal activity of the aqueous stem extract of Lantana camara Linn (Verbenaceae) in mice. BMC Complementary and Alternative Medicine, 17(1), 190–190. 10.1186/s12906-017-1696-1 28376868 PMC5379525

[fsn31745-bib-0030] Tasev, K. , Stefova, M. , & Ivanova, V. (2016). HPLC method validation and application for organic acid analysis in wine after solid‐phase extraction. Macedonian Journal of Chemistry and Chemical Engineering, 35(2), 225–233. 10.20450/mjcce.2016.1073

[fsn31745-bib-0031] Tesfay, B. , Mulugeta, G. , & Tadesse, A. (2011). Description of cactus pear (opuntia ficus‐indica (l.) mill.) cultivars from Tigray, northern Ethiopia. Mekelle. Tigray, Ethiopia: Tigray: Agricultural Research Institute. 10.20450/mjcce.2016.1073

[fsn31745-bib-0032] Verón, H. E. , Cano, P. G. , Fabersani, E. , Sanz, Y. , Isla, M. I. , Espinar, M. T. F. , … Torres, S. (2019). Cactus pear (Opuntia ficus‐indica) juice fermented with autochthonous Lactobacillus plantarum S‐811. Food & Function, 10(2), 1085–1097. 10.1039/c8fo01591k 30720817

[fsn31745-bib-0033] Vilela, A. (2019). Use of nonconventional yeasts for modulating wine acidity. Fermentation, 5(1), 27 10.3390/fermentation5010027

[fsn31745-bib-0034] Volschenk, H. , Van Vuuren, H. , & Viljoen‐Bloom, M. (2006). Malic acid in wine: Origin, function and metabolism during vinification. South African Journal of Enology & Viticulture., 27(2), 123–136. 10.21548/27-2-1613

[fsn31745-bib-0035] Yolmeh, M. , & Jafari, S. M. (2017). Applications of response surface methodology in the food industry processes. Food and Bioprocess Technology, 10(3), 413–433. 10.1007/s11947-016-1855-2

[fsn31745-bib-0036] Yusufu, M. , Pg, J. , & Sa, A. (2018). Production and quality evaluation of wine from watermelon juice and ginger extract. Journal of Human Nutrition & Food Science, 6(1), 1122.

[fsn31745-bib-0037] Zenebe, T. T. , Chanukya, S. , & Solomon, M. L. (2018). Optimization of cactus pear fruit fermentation process for wine production. Foods, 7(8), 121 10.3390/foods7080121 PMC6111886 30061505

[fsn31745-bib-0038] Zenebe, T. T. , & Kidu, M. G. (2019). Physicochemical and sensory properties of wine produced from blended Cactus Pear (Opuntia ficus‐indica) and Lantana camara (L. camara) Fruits. Journal of Food Quality, 2019 10.1155/2019/6834946

[fsn31745-bib-0039] Zenebe, T. T. , & Solomon, M. L. (2020). Response surface optimization of Cactus Pear (Opuntia ficus‐indica) with Lantana camara (L. camara) fruit fermentation process for quality wine production. International Journal of Food Science, 2020 10.1155/2020/8647262 PMC7201468 32399478

[fsn31745-bib-0040] Zhong, W. , Chen, T. , Yang, H. , & Li, E. (2020). Isolation and selection of non‐saccharomyces yeasts being capable of degrading citric acid and evaluation its effect on kiwifruit wine fermentation. Fermentation, 6(1), 25 10.3390/fermentation6010025

